# Impact of the B Cell Growth Factor APRIL on the Qualitative and Immunological Characteristics of Atherosclerotic Plaques

**DOI:** 10.1371/journal.pone.0164690

**Published:** 2016-11-07

**Authors:** Sophie J. Bernelot Moens, Sander I. van Leuven, Kang H. Zheng, Stefan R. Havik, Miranda V. Versloot, Leonie M. van Duivenvoorde, Michael Hahne, Erik S. G. Stroes, Dominique L. Baeten, Anouk A. J. Hamers

**Affiliations:** 1 Department of Vascular Medicine, Academic Medical Center, Amsterdam, The Netherlands; 2 Amsterdam Rheumatology and Immunology Center, Department of Clinical Immunology and Rheumatology, Academic Medical Center, Amsterdam, The Netherlands; 3 Department of Experimental Vascular Medicine, Academic Medical Center, Amsterdam, The Netherlands; 4 Department of Experimental Immunology, Academic Medical Center, Amsterdam, The Netherlands; 5 Institut de Génétique Moléculaire de Montpellier, Centre National de la Recherche Scientifique, Université de Montpellier, Montpellier, France; Max Delbruck Centrum fur Molekulare Medizin Berlin Buch, GERMANY

## Abstract

Studies on the role of B lymphocytes in atherosclerosis development, have yielded contradictory results. Whereas B lymphocyte-deficiency aggravates atherosclerosis in mice; depletion of mature B lymphocytes reduces atherosclerosis. These observations led to the notion that distinct B lymphocyte subsets have different roles. B1a lymphocytes exert an atheroprotective effect, which has been attributed to secretion of IgM, which can be deposited in atherosclerotic lesions thereby reducing necrotic core formation. Tumor necrosis factor (TNF)-family member ‘A Proliferation-Inducing Ligand’ (APRIL, also known as TNFSF13) was previously shown to increase serum IgM levels in a murine model. In this study, we investigated the effect of APRIL overexpression on advanced lesion formation and composition, IgM production and B cell phenotype. We crossed APRIL transgenic (APRIL-Tg) mice with ApoE knockout (ApoE^-/-^) mice. After a 12-week Western Type Diet, ApoE^-/-^APRIL-Tg mice and ApoE^-/-^ littermates showed similar increases in body weight and lipid levels. Histologic evaluation showed no differences in lesion size, stage or necrotic area. However, smooth muscle cell (α-actin stain) content was increased in ApoE^-/-^APRIL-Tg mice, implying more stable lesions. In addition, increases in both plaque IgM deposition and plasma IgM levels were found in ApoE^-/-^APRIL-Tg mice compared with ApoE^-/-^ mice. Flow cytometry revealed a concomitant increase in peritoneal B1a lymphocytes in ApoE^-/-^APRIL-Tg mice. This study shows that ApoE^-/-^APRIL-Tg mice have increased oxLDL-specific serum IgM levels, potentially mediated via an increase in B1a lymphocytes. Although no differences in lesion size were found, transgenic ApoE^-/-^APRIL-Tg mice do show potential plaque stabilizing features in advanced atherosclerotic lesions.

## Introduction

With the increasing recognition of atherosclerosis as an inflammatory disease [1], understanding the role of different immune cells is pivotal for therapeutic targeting of the inflammatory process during atherogenesis. Amongst the immune cells involved in atherosclerosis, studies on the role of B lymphocytes have yielded contradictory results. In humans, early data suggesting a role for B lymphocytes in atherosclerosis was derived from a study in war veterans, showing that trauma-induced splenectomy is associated with a high rate of acute myocardial infarction [2]. This was corroborated by genome wide association data, supporting a protective role of B lymphocytes in atherosclerosis [3].

In murine models, however, conflicting results have been found. B lymphocyte-deficient mice had accelerated atherosclerosis [4]. Conversely, mature B lymphocyte depletion by anti-CD20 antibodies reduced atherosclerosis [5,6]. These observations led to the notion of distinct B lymphocyte subsets with different roles. Whereas B2 lymphocytes seem to aggravate atherosclerosis [5], B1a lymphocytes have atheroprotective properties [7], potentially through secretion of natural IgM [8], which can be deposited in atherosclerotic lesions. IgM is thought to play a role in the clearance of oxidized LDL (OxLDL) and apoptotic cells [9], thereby reducing the necrotic core [8]. Low IgM levels in general (independent of their specific epitope) are associated with increased lesion formation [10], and infusion of polyclonal IgM reduced acceleration of lesion progression in mice [11], supporting that overall increases in IgM levels have atheroprotective properties. Also in humans, IgM antibodies have been shown to have an inverse correlation to carotid atherosclerosis [12] and CVD risk [13].

The tumor necrosis factor (TNF)-family members B-cell activating factor (BAFF) and ‘A Proliferation-Inducing Ligand’ (APRIL, also known as TNFSF13) [14] are critical regulators of B cell homeostasis [15]. In murine models of collagen induced arthritis BAFF and APRIL are suggested to have opposite effects: BAFF promotes inflammatory processes, whereas overexpression of APRIL suppressed experimental arthritis, potentially mediated via selective increases in plasma IgM levels [16]. In atherogenesis, BAFF receptor-deficiency was shown to decrease atherosclerosis, by depleting B2 but not B1a lymphocytes [7,17]. Both RNA and protein expression of BAFF and APRIL have been shown in human atherosclerotic tissue and plasma [18,19]. The role of APRIL in atherosclerosis has not yet been investigated. In the present study, we hypothesized that ectopic APRIL expression on an atherosclerotic background (ApoE knockout mice), would reduce atherosclerotic lesion formation through increased IgM production.

## Methods

### Animals and Experimental Design

Heterozygous APRIL-Tg C57BL/6.J mice, which express human APRIL under control of the LCK distal promoter (as described in detail previously), [16] (kindly provided by Dr. M. Hahne) were crossed with ApoE knockout (ApoE^-/-^) C57BL/6.J mice (purchased from Charles River, Jackson Laboratories, Bar Harbor, Maine). Genotype was established by polymerase chain reaction on DNA isolated from the toe. The ApoE-specific primer set was: forward 5’GCCTAGCCGAGGGAGAGCCG-3’; Wild type reverse 5’-TGTGACTTGGGAGCTCTGCAGC-3’; Mutant reverse 5’-GCCGCCCCGACTGCATCT-3’. The APRIL-specific primer set was: forward 5’-ATGGATTACAAAGACGATGACG-3’ and reverse 5’-TCACAGTTTCACA AACCCCAGG-3’. 12–14 Weeks old female ApoE^-/-^APRIL-Tg mice and ApoE^-/-^ littermates were fed a Western Type Diet (WTD, Abdiets, Woerden, the Netherlands) containing 0.25% (w/w) cholesterol and 16% (w/w) fat for 12 weeks. Body weight was determined every week. Blood samples (tail snip) were taken at t = 0 and t = 6 weeks after a 4 hour fasting period. At t = 12 weeks mice were first fasted for 4 hours and sacrificed by an intra-peritoneal ketamine (238 mg/kg) / xylazine (24 mg/kg) injection. Blood was collected through orbital bleeding and plasma was stored at -20°C until use. Peritoneal lavage was performed with 5ml of ice-cold phosphate-buffered saline (PBS; Fresenius Kabi, Zeist, The Netherlands). Hearts were cut perpendicular to the heart axis just below the atrial tips, embedded in paraffin and 7μM sections were made. Aorta’s and Lymph nodes were snap frozen. Animal experiments were performed in compliance with Dutch national and institutional guidelines and approved by the Committee for Animal Welfare of Amsterdam Medical Centre (Permit Number DRI102945). All efforts were made to minimize animal suffering. More detailed information on experimental procedures, animals and housing is available in **S1 Text**.

### Mouse blood parameters

White blood cells (WBC), Red blood cells (RBC), platelets and hematocrit (Hct) were measured using a ScilVet abc plus+ (ScilVet, Oostelbeers, The Netherlands). Plasma triglyceride levels were measured colorimetrically (GPO-PAP, Roche, Woerden, The Netherlands) and plasma total cholesterol was determined enzymatically (CHOD-PAP, Roche, Woerden, The Netherlands) according to the manufacturer's instructions. Total plasma IgM and IgG levels were determined by using a standard ELISA technique. Briefly, plates were coated overnight at 4°C with goat anti-mouse Ig(H+L) (Southern Biotech, Birmingham, LA, USA). Plasma samples were incubated for 2 hours at room temperature followed by a goat-anti-mouse IgM-HRP or goat anti-mouse IgG(H+L) human ads-HRP (Southern Biotech, Birmingham, LA, USA). IgM and IgG levels were visualized by using ABTS Elisa peroxidase substrate (2,5 mg/ml in 0.1M Citrate-phosphate buffer) and measured at 405 nm on a VersaMax microplate reader (Molecular Devices, Sunnyvale, CA, USA). Specific antibody titers against Cu_2_SO_4_-oxidized LDL (CuOx-LDL) and malondialdehyde-modified LDL (MDA-LDL) were determined as described previously [20].

### Flow Cytometry

Peritoneal and splenic B lymphocyte subsets were analyzed on a BD FACS Canto II flow cytometer (Becton, Dickinson, Franklin Lakes, NJ, USA) by using the following antibodies: CD19-FITC, CD5-PerCPcy5.5 and CD11b-A700 (all from eBioscience, Vienna, Austria). Lymphocytes were gated in the forward/side scatter and B lymphocytes were classified according to CD19 expression and further divided into CD5^+^ B1a lymphocytes [8], CD5^-^CD11b^+^ B1b lymphocytes, and CD5^-^CD11b^-^ B2 lymphocytes [21]. Samples were analyzed using FlowJo software version 7.6.5. (FlowJo, LLC, Ashland, OR, USA).

### Gene expression

From blood cells Total RNA was extracted using the Aurum^TM^ Total RNA Mini Kit (BioRad, Hercules, CA, USA) and for the snap frozen Lymph nodes and aorta’s total RNA was extracted after crushing (under liquid nitrogen) using Trizol (Life technologies). cDNA was made from 500ng total RNA using iScript cDNA Synthesis kit (BioRad). Semi-quantitative real-time PCR was performed using iQ SYBR Green Supermix (BioRad) and was measured with the MyIQ system. The following primers were designed for CD19 and Rplp0 (to correct for cDNA content) were designed (**S1 Table**). A TaqMan assay was done using TaqMan Gene Expression MasterMix (Thermo Fisher Scientific, Waltham, MA, USA) and was measured on a StepOnePlus^TM^ Real-Time PCR system (Applied Biosystems; Thermo Fisher Scientific). APRIL and HPRT probes were purchased from Thermo Fisher Scientific (**S1 Table**).

### Cytochemical and immunohistological stainings

Paraffin sections were deparaffinized and rehydrated. Aortic root lesions were visualized using hematoxylin-eosin (H&E) stain. Plaque size and necrotic core were quantified using Adobe photoshop CS5 software. Plaque stage was determined according to the method described by de Waard et al. [22]. For further plaque phenotyping, aortic roots were stained for 60 minutes with a 0.2% Picro Sirius Red solution and incubated for 2 minutes in acidified water (0.01M HCl). The sections were dehydrated, embedded in pertex (Histolab, Västra Frölunda, Sweden) and collagen content was quantified using Adobe photoshop CS5 software. In addition, sections were incubated with antibodies detecting macrophages (MAC-3; BD Pharmingen, San Jose, CA, USA), smooth muscle cells (SMCs; 1A4; Dako, Glostrup, Denmark), and IgM (polyclonal; Abcam, Cambridge, MA, USA) followed by a horseradish peroxidase (HRP)-conjugated secondary antibody. DAB substrate (ImmunoLogic, Duiven, The Netherlands) was used for detection. After counterstaining with hematoxylin sections were embedded in pertex. Finally, the positive stained area was quantified in 4 sections per mouse using Adobe Photoshop CS5 software and calculated as a percentage from total lesion size.

### Statistical analyses

Data are presented as mean±SEM, median (IQR) or n(%). Unpaired t-tests or Mann Whitney U tests were used to assess differences between the 2 groups depending on a normal distribution. Differences in plaque stage were assessed using a Chi square test. All data were analysed using Prism version 5.0 (GraphPad software, La Jolla, CA, USA) and SPSS version 22.0 (SPSS Inc., Chicago, Il, USA). A p<0.05 was considered statistically significant.

## Results

### APRIL overexpression does not affect lesion size

**Fig 1:**
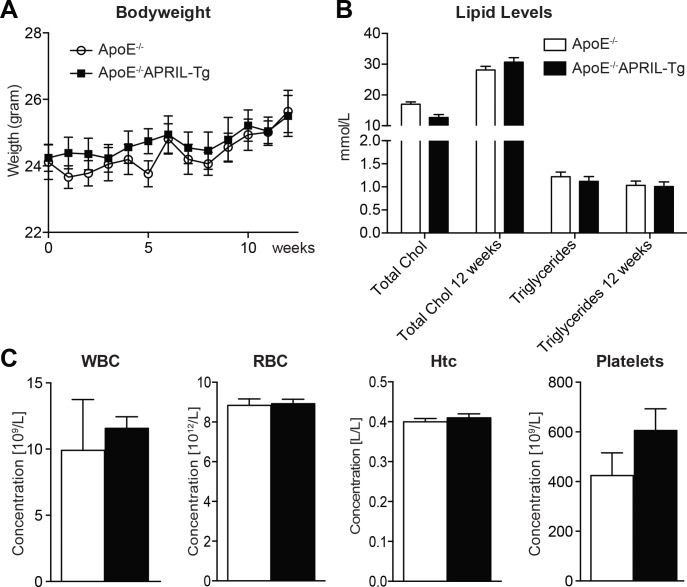
Bodyweight, lipid levels and blood cell counts of ApoE^-/-^ and ApoE^-/-^APRIL-Tg mice. ApoE^-/-^ (n = 13) and ApoE^-/-^APRIL-Tg mice (n = 10). Every week body weight was determined (A), plasma cholesterol and triglycerides were measured before the start of diet and after 12 weeks of WTD (B). After 12 weeks of WTD the number of blood cells were measured by a cell counter (C). Data are represented as mean±SEM. Chol (cholesterol); WBC (white blood cells); RBC (red blood cells); Htc (hematocrit).

**Fig 2:**
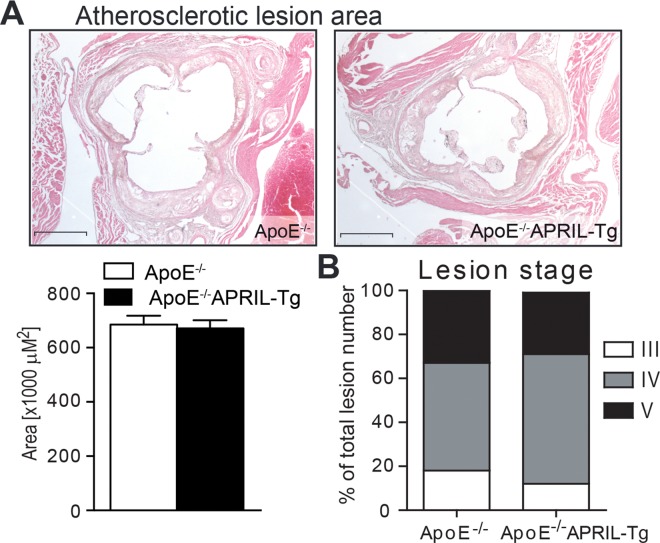
Lesion size and stage of ApoE^-/-^ and ApoE^-/-^APRIL-Tg mice. After 12 weeks of WTD lesion size (A) was quantified and lesion stage (B) was determined in the aortic roots of ApoE^-/-^ (n = 13) and ApoE^-/-^APRIL-Tg mice (n = 10). Representative photomicrographs are shown with original magnification x25. Data are represented as mean±SEM; Scale bars represent 1mm.

ApoE^-/-^APRIL-Tg mice show normal growth, are viable and fertile. APRIL mRNA expression is significantly increased in the lymph nodes, but no increase is found in the blood compartment or abdominal aorta (S1 Fig). During the course of a 12-week WTD, ApoE^-/-^ (n = 13) and ApoE^-/-^APRIL-Tg (n = 10) mice showed comparable increments in bodyweight and plasma lipid levels (**Fig 1A and 1B**). Hematological blood parameters were also similar between the two groups (**Fig 1C**). Assessing the aortic roots, there was no difference in total lesion area (**Fig 2A**) with the majority of mice in both groups showing advanced atherosclerotic lesions (82% in stage IV or V in ApoE^-/-^ mice and 87% in ApoE^-/-^APRIL-Tg, p = 0.2821) (**Fig 2B**).

### APRIL overexpression increases plaque smooth muscle cell content

**Fig 3:**
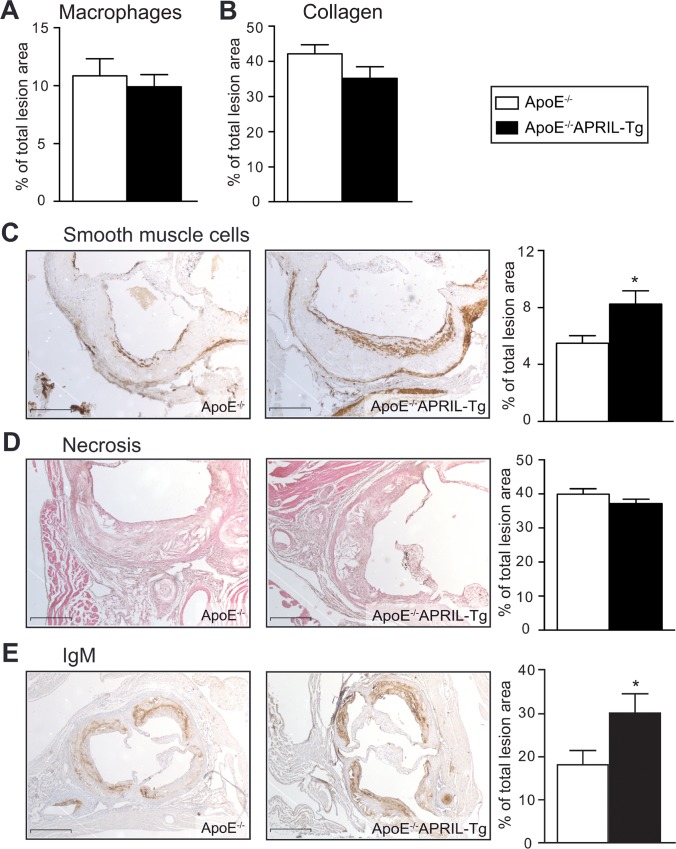
Lesion characteristics of ApoE^-/-^ and ApoE^-/-^APRIL-Tg mice. ApoE^-/-^ (n = 13) and ApoE^-/-^APRIL-Tg mice (n = 10). After 12 weeks of WTD macrophage content (A) and the percentage of collagen deposition (B) were quantified. Representative photomicrographs and quantification of Smooth muscle cell content (C), necrosis (D), and IgM deposition (E) are shown. Original magnification x50 (C+D) and x25 (E). Data are represented as mean±SEM; *p<0.05; Scale bars represent 1mm.

Since lesion composition is equally important as lesion size in predicting cardiovascular outcome, we sought to investigate lesion cellular composition as well as necrotic area. In the current study, we observed no significant differences in macrophage content, collagen deposition or necrotic core area between the 2 groups (**Fig 3A–3C**). However, SMC content was significantly increased in ApoE^-/-^APRIL-Tg mice (8.2%±0.9 vs 5.5%±0.5 in ApoE^-/-^, p = 0.0143) (**Fig 3D**) indicating that the lesions may more stable in APRIL-Tg mice.

### APRIL overexpression increases IgM levels and peritoneal B1a cells

**Fig 4:**
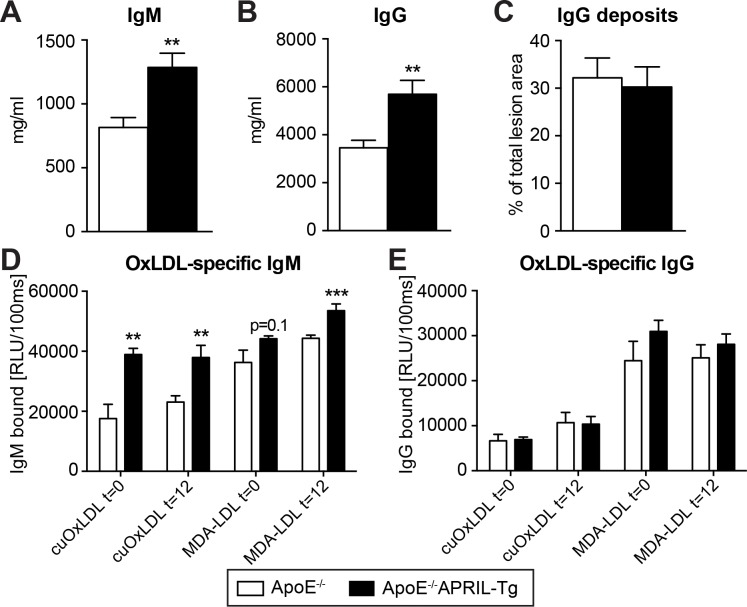
Immunoglobulins of ApoE^-/-^ and ApoE^-/-^APRIL-Tg mice. After 12 weeks WTD both plasma IgM (A) and IgG (B) were measured. In ApoE^-/-^ (n = 13) and ApoE^-/-^APRIL-Tg mice (n = 10) IgG deposition was quantified as a % of total lesion size (C). Specific antibodies to copper-oxidized (CuOx) or malondialdehyde (MDA)-modified LDL (quantified as relative light units (RLU)) were determined for both IgM (**D**) and IgG (**E**) before start of the WTD and at harvest (after 12 weeks WTD). F, percentage of CD19^+^ cells, as well as the percentages of each subset are shown. Data are represented as mean±SEM; **p<0.01 ***p<0.001.

**Fig 5:**
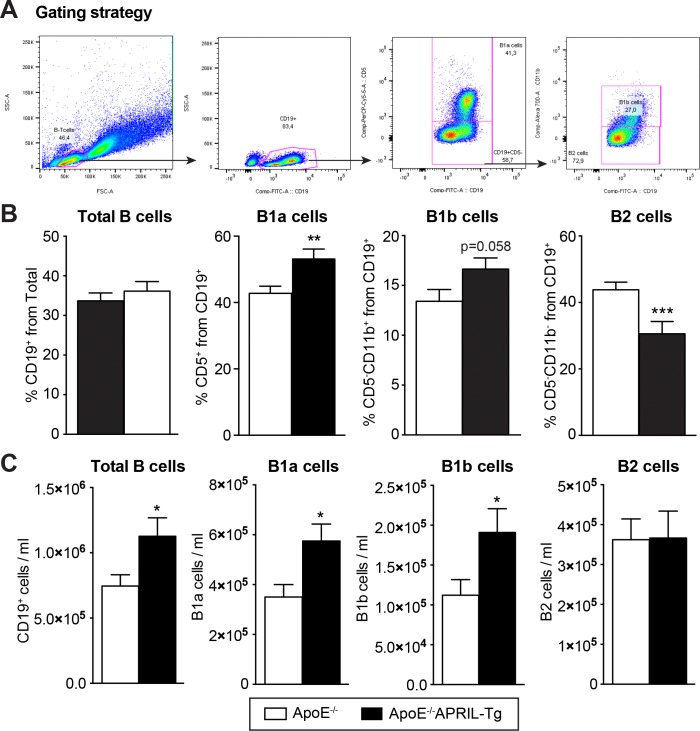
Peritoneal B cell subsets of ApoE^-/-^ and ApoE^-/-^APRIL-Tg mice. Peritoneal B cell subsets were quantified by FACS analysis after 12 weeks of WTD in ApoE^-/-^ (n = 13) and ApoE^-/-^APRIL-Tg mice (n = 10). The cells were gated (A) for Lymphocytes in the FSC/SSC plot and B cells were selected on the basis of CD19 positivity. Subsets were identified as follows: CD5^+^CD11b^+^B1a cells, CD5^-^CD11b^+^ B1b cells, and CD5^-^CD11b- B2 cells. The percentages (B) and concentration (C) of CD19^+^ cells, as well as for each B cell subset are shown. Data are represented as mean±SEM; *p<0.05 **p<0.01 ***p<0.001.

Considering previous reports demonstrating that APRIL overexpression increases IgM [16], we assessed IgM levels in both the lesions and plasma. Lesion IgM content was almost 2-fold higher in ApoE^-/-^APRIL-Tg mice compared to ApoE^-/-^ mice (30%±5 and 18%±4, respectively; p = 0.0479) (**Fig 3E**). In addition, plasma IgM levels were increased 1.6 fold (ApoE^-/-^: 815±77 μg/ml, ApoE^-/-^APRIL-Tg: 1286±110 μg/ml, p = 0.0015) (**Fig 4A**). Unexpectedly, plasma IgG levels were also augmented in the ApoE^-/-^APRIL-Tg mice in comparison to controls (5692±575 μg/ml and 3459±310 μg/ml, respectively; p = 0.0017) (**Fig 4B**), however, this did not translate to an increase in lesion deposition (32%±15 in ApoE^-/-^ versus 30%±13 in ApoE^-/-^ APRIL-Tg, p = 0.753) (**Fig 4C**). Further specifying plasma IgM and IgG increases we found that the IgM increase corresponded with a significant increase in anti-cuOx-LDL and anti-MDA-LDL antibodies, whereas the increase in IgG did not (**Fig 4D and 4E**). Since B1a lymphocytes are potent IgM producers [8] and B2 lymphocytes are known for their IgG production [23], we assessed the peritoneal B lymphocyte subsets. Flow cytometry revealed that the total percentage of B lymphocytes was comparable between ApoE^-/-^APRIL-Tg mice and ApoE^-/-^ mice (36±2 versus 34±2, respectively). Moreover, total CD19 expression in the blood, lymph nodes and aorta showed were similar; suggesting no differences in the amount of B cells present (**S2A Fig**). We did find a 24% elevation in B1a (CD19^+^CD5^+^) lymphocytes in ApoE^-/-^APRIL-Tg mice (53%±3) compared to the ApoE^-/-^ (43%±2, p = 0.01) (**Fig 5A and 5B**). B1b cells (CD5^-^CD11b^+^) cells were also moderately increased (ApoE^-/-^APRIL-Tg mice 13±1% versus ApoE^-/-^ 17±1%, p = 0.06). The atherogenic B2 lymphocyte subset (CD5^-^CD11b^-^) however, showed a relative decrease in ApoE^-/-^APRIL-Tg mice (31±3%) compared to ApoE^-/-^ mice (44±2%, p = 0.0009). To assess whether these changes reflected absolute differences, we used cell concentrations of peritoneal lavage fluid to calculate CD19^+^ cell concentration, revealing that the total number of B cells in the peritoneal cavity, was in fact significantly increased compared to ApoE^-/-^ (p = 0.03). Moreover, the increase of B1 cells cannot be addressed to a decrease in B2 cells (**Fig 5C**). As expected, we found a similar splenic B lymphocyte subset distribution in both groups, since B1 cells mainly reside in serosal cavities (such as the peritoneum) and are only a minor population in the spleen [24] (**S2B Fig**).

## Discussion

The present study used a novel mouse model to investigate the effect of transgenic overexpression of APRIL on an ApoE^-/-^ background regarding 1) atherosclerotic lesion progression; 2) IgM production; and 3) B lymphocyte phenotype. We show that in this model, IgM levels were increased 1.5 fold; potentially mediated via an increase in B1a lymphocyte numbers. This increase did not affect lesion size or stage, but we did observe phenotypical changes of the atherosclerotic lesion, with increases in smooth muscle cell numbers accompanied by an unchanged macrophage content.

APRIL overexpression was associated with a significant increase of total plasma IgM levels, as well as specific IgM antibodies against OxLDL, and a concomitant increase in plaque deposition of IgM, which coincided with a significant increase in B1a lymphocytes. The APRIL transgenic mice have been described in detail previously [16]. Briefly, expression of human APRIL under the control of the Lck distal promoter directs transgene expression to immature thymocytes and peripheral T lymphocytes; after which it can bind two members of the TNF receptor family: the transmembrane activator and calcium modulator cyclophilin ligand interactor (TACI) and B cell maturation antigen (BCMA), involved in B and T cell homeostasis and activation [15]. Importantly, in line with data on experimental arthritis [25], APRIL-Tg mice do not show the severe adverse effects associated with BAFF receptor signaling (the other ligand for BCMA and TACI) [7,17], exhibiting significantly lower percentages of the atherogenic B2 lymphocyte subset. However, we could not corroborate previously reported beneficial effects of IgM on atherosclerotic lesion size and composition, including a more stable phenotype with less necrosis [7,8,11,26]. Several explanations could contribute to the absence of such a beneficial effect in our model. In two of the previously mentioned studies, absolute differences in plasma IgM and plaque IgM were larger compared to the present study. Kyaw and colleagues [7] reported an 80% decrease in plasma IgM levels in BAFF-R deficient mice, and another group showed that adoptive transfer of B1a lymphocytes in splenectomized mice led to a 68% increase in plasma IgM [26]. In our study an overall 57% increase was found in plasma levels, however, it has also been reported that in APRIL-TG mice, B1 cell expansion and IgM production accumulate over time [27]. This suggests that, whereas in the previously mentioned studies levels of IgM were stable throughout the experiments, in our model levels may have been lower in the earlier stages of atherogenesis, supported by the finding that indeed specific antibodies against MDA-LDL increased over the course of the experiment.

The observed increase in total B cell numbers was not expected with APRIL overexpression [25]. Interestingly, Rincón-Arévalo and colleagues [28] recently reported that dyslipidemia following a high fat diet is capable of increasing total B lymphocytes (while lowering B lymphocyte CD19 expression) and concomitantly increasing IgG1 serum levels, attributed to intra-cellular lipid accumulation. We also found an unexpected increase in total serum IgG in ApoE^-/-^APRIL-Tg mice [25]. Recently, lipid accumulation has also been described to activate human monocytes [29], supporting detrimental effects of continuous high blood lipid levels on the immune system. Whether the WTD may have influenced the increased cell numbers and IgG serum levels found in ApoE^-/-^APRIL-Tg mice compared to ApoE^-/-^, was not addressed in this study. However, it should be noted that the increase in total IgG levels did not result in any increase of IgG antibodies against OxLDL or increased IgG deposition in atherosclerotic lesions. The role of IgG antibodies in atherosclerosis is still poorly understood and both protective and detrimental effects have been described [23]; therefore it remains elusive whether the increase in IgG contributed to the lack of effect in our study.

Finally, in contrast to a previous study which reported that serum IgM-deficient LDLR^-/-^ mice demonstrated a significantly accelerated atherosclerosis with increased plaque SMC content [10], we found increased SMC content with higher IgM levels. Apoptosis of smooth muscle cells is considered an important feature of fibrous cap thinning, which in turn is an important marker for plaque vulnerability and destabilization [30], eventually contributing to the risk of rupture and subsequent CV events [31]. Increasing SMCs, has been previously associated with a more stable-appearing phenotype [32].

Overall, we find that overexpression of APRIL on an atherogenic background increases plasma IgM levels and plaque deposition. Although we did not find an important role for ectopic overexpression of APRIL regarding plaque size, the phenotypical remodeling of advanced atherosclerotic lesion warrants future research to the underlying mechanisms and its consequences for cardiovascular disease.

## Supporting Information

S1 ARRIVE Checklist (PDF)

S1 Fig APRIL expression. Taqman assays were performed in blood cells, …. Lymph nodes and aorta’s of ApoE^-/-^ (n = 13) and ApoE^-/-^APRIL-Tg mice (n = 10) after 12 weeks of WTD. Data are represented as mean±SD. *p<0.05.
(EPS)

S2 Fig B cell numbers in different compartments. After 12 weeks of WTD, a qRT-PCR for CD19 expression (A) was performed on total blood cells, lymph nodes, and aorta from ApoE^-/-^ (n = 13) and ApoE^-/-^APRIL-Tg mice (n = 10) (A). Splenic B cell subsets were quantified by FACS analysis as a percentage from total CD19+ cells (B). Subsets were identified as follows: CD5^+^CD11b^+^B1a cells, CD5^-^CD11b^+^ B1b cells, and CD5^-^CD11b- B2 cells. Data are represented as mean±SD (A) or mean±SEM (B).
(EPS)

S1 File SPSS raw data. Raw data used for the article.
(SAV)

S1 Table Primer sequences and TaqMan probes. (PDF)

S1 Text Supplemental Materials and Methods. (PDF)
