# Supplementary material for: Impact of the B Cell Growth Factor APRIL on the Qualitative and Immunological Characteristics of Atherosclerotic Plaques
Source: PLoS One. 2016 Nov 7;11(11):e0164690. doi: 10.1371/journal.pone.0164690 (PMC5098816; doi:10.1371/journal.pone.0164690)
Supplement: S1 Table — (PDF) [file pone.0164690.s005.pdf]

S1 Table. Primer sequences and TaqMan probes.

Gene	Forward primer sequence	Reverse primer sequence
Rplp0	5’-GGACCCGAGAAGACCTCCTT-3’	5’-GCACATCACTCAGAATTTCAATGG-3’
CD19	5’-AGGATGATGGACTTCCTGAGC-3’	5’-ATGACTGGGACCGGACTGAA-3’
APRIL[a]	Hs00182565_m1	
HPRT[a]	Mm00446968_m1	

[a] indicates TaqMan Probes
