# Supplementary material for: Impact of the B Cell Growth Factor APRIL on the Qualitative and Immunological Characteristics of Atherosclerotic Plaques
Source: PLoS One. 2016 Nov 7;11(11):e0164690. doi: 10.1371/journal.pone.0164690 (PMC5098816; doi:10.1371/journal.pone.0164690)
Supplement: S1 Text — (PDF) [file pone.0164690.s006.pdf]

Supplemental Materials and Methods.

Study design and experimental procedures.

starting the experimental procedures sample size necessary in order to detect a 20% difference in primary outcome was calculated. We needed 15 animals to have sufficient power to detect a 20% difference with a power of 80% and a p-value of 0.05. Primary outcome of the atherosclerosis model was the atherosclerotic lesion size. Secondary, the cellular composition of the lesion was assessed by immunohistochemistry.

During our experiments mice were weighed every week at exactly the same day and time to monitor their weight gain. In addition, all animals in the experiments were evaluated by a welfare diary to see whether any deviations from normal behavior occur such as: fur, posture, activity and skin. One $ApoE^{-/-}$APRIL-Tg mouse died at the start of WTD. At the end of the experiment, mice were sacrificed by an intraperitoneal ketamine/xylazine injection depending on the weight of the mice (ketamine = 238mg/kg, xylazine = 24mg/kg).

Housing.

The mice were housed in standard cages at the specific pathogen free (SPF) animal facility of the Amsterdam Medical Centre. At this facility the light dark cycle (12:12) was strictly regulated and the ambient temperature was kept between 20 and 25°C. Animals were kept in social groups from 2 to 6 individuals per cage and were identified by ear marks. The bedding material consisted of autoclaved hardwood material and tissue paper was used as nesting material. Also, a nesting box was provided. Mice had ad libitum access to food (Western type diet;) and slightly acidified water.
